# Associations of Unilateral Whisker and Olfactory Signals Induce Synapse Formation and Memory Cell Recruitment in Bilateral Barrel Cortices: Cellular Mechanism for Unilateral Training Toward Bilateral Memory

**DOI:** 10.3389/fncel.2016.00285

**Published:** 2016-12-16

**Authors:** Zilong Gao, Lei Chen, Ruicheng Fan, Wei Lu, Dangui Wang, Shan Cui, Li Huang, Shidi Zhao, Sudong Guan, Yan Zhu, Jin-Hui Wang

**Affiliations:** ^1^State Key Lab of Brain and Cognitive Sciences, Institute of Biophysics, Chinese Academy of SciencesBeijing, China; ^2^College of Life Sciences, University of Chinese Academy of SciencesBeijing, China; ^3^Department of Pathophysiology, Bengbu Medical CollegeBengbu, China; ^4^School of Pharmacy, Qingdao UniversityShandong, China

**Keywords:** memory, glutamate, GABA, neuron, synapse, barrel cortex, whisker, olfaction

## Abstract

Somatosensory signals and operative skills learned by unilateral limbs can be retrieved bilaterally. In terms of cellular mechanism underlying this unilateral learning toward bilateral memory, we hypothesized that associative memory cells in bilateral cortices and synapse innervations between them were produced. In the examination of this hypothesis, we have observed that paired unilateral whisker and odor stimulations led to odorant-induced whisker motions in bilateral sides, which were attenuated by inhibiting the activity of barrel cortices. In the mice that showed bilateral cross-modal responses, the neurons in both sides of barrel cortices became to encode this new odor signal alongside the innate whisker signal. Axon projections and synapse formations from the barrel cortex, which was co-activated with the piriform cortex, toward its contralateral barrel cortex (CBC) were upregulated. Glutamatergic synaptic transmission in bilateral barrel cortices was upregulated and GABAergic synaptic transmission was downregulated. The associative activations of the sensory cortices facilitate new axon projection, glutamatergic synapse formation and GABAergic synapse downregulation, which drive the neurons to be recruited as associative memory cells in the bilateral cortices. Our data reveal the productions of associative memory cells and synapse innervations in bilateral sensory cortices for unilateral training toward bilateral memory.

## Introduction

Associative memory is essential for the cognitions (Wasserman and Miller, 1997; Suzuki, 2008; Lansner, 2009). After somatosensory signals and operative skills are learnt by unilateral limbs, these signals and skills can be retrieved and operated in bilateral limbs. Operative ability and precision are greater in the training side than contralateral side. This signal transfer from unilateral learning to bilateral memory is essential for bilateral limbs to coordinately handle environment changes, in which the corpus callosum may be required since it connects bilateral hemispheres (Witelson, 1985; Dubb et al., 2003; Hofer and Frahm, 2006), coordinates bilateral limbs’ activities (Caeyenberghs et al., 2011; Lum et al., 2011; Gooijers and Swinnen, 2014) and contributes to intellectual processes (Piercy, 1967; Clark and Geffen, 1989; Hasegawa et al., 1998; Hasegawa, 2000; Harris et al., 2001; Kozlovskiy et al., 2012). In addition to the corpus callosum for signal transfer bilaterally, comprehensive picture for unilateral learning toward bilateral memory should include the memory cells in both sides of the cerebral cortices for information storage. Potential cellular mechanisms remain to be examined, such as the upregulation of innate bilateral connections, the formation of new synapse innervations from the training side to its contralateral side and the production of memory cells in bilateral cortices.

There are minor connections between bilateral somatosensory cortices in adult animals (Olavarria et al., 1984; Aronoff et al., 2010), which may be inter-hemisphere inhibition (Kawaguchi, 1992; Shuler et al., 2001; Glazewski et al., 2007) or less interactions (Armstrong-James and George, 1988). This feature supports a fact that sensory signals from unilateral limbs cannot be felt in contralateral limbs. In this regard, the somatosensory cortex, which may be involved in information storage (Diamond et al., 2003; Diamond and Arabzadeh, 2013), would be ideally used to study the recruitment of bilateral connections for unilateral learning toward bilateral memory, instead of the studies by using special sensory cortices in that splitting hemispheres is needed. After associative memory is onset in sensory cortices (Weinberger, 2004, 2007; Letzkus et al., 2012; Wang et al., 2015), the nerve cells in the trained somatosensory cortices can be recruited as associative memory cells (Wang et al., 2014, 2015, 2016). These associative memory cells hypothetically send the newly learnt sensory signal to the neurons in the contralateral somatosensory cortex by their axon projections and new synapse innervations, such that the neurons in the contralateral cortex are recruited as associative memory cells for unilateral training toward bilateral memory.

Associative learning is a common way for the information acquisition. Classical conditionings, such as fear conditioning (Davis et al., 1993; Reijmers et al., 2007; Maren, 2008; Perkowski and Murphy, 2011) and eye-blinking conditioning (Burhans et al., 2008; Woodruff-Pak and Disterhoft, 2008; Bracha et al., 2009), are applied to elucidate the mechanisms underlying associative memory. These studies have not paid attention to unilateral training toward bilateral memory. Current reports indicate that pair-stimulations to unilateral whiskers and olfaction lead to odorant-induced whisker motion in the mice and that their barrel cortex becomes able to encode both whisker and odor signals (Wang et al., 2013, 2014, 2015). With this model, we aim to examine whether unilateral training can induce bilateral memory and how the neurons in both sides of the barrel cortices are recruited to be associative memory cells for this process, especially the formation of synapse innervations between bilateral barrel cortices as well as the refinements of glutamatergic and GABAergic neurons. In terms of strategies to test the hypotheses above, pAAV-SynaptoTag-mCherry-green fluorescent protein (GFP) was injected into the trained barrel cortex for tracing axon projection and synapse formation in its contralateral barrel cortex (CBC) by cellular imaging. Electrophysiological recording in bilateral barrel cortices *in vivo* was used to analyze how the neurons encode these associated signals. Whole-cell recordings in the brain slices were used to assess the refinement of the neurons and synapses.

## Materials and Methods

All experiments were performed in accordance with the guidelines by the Administration Office of Laboratory Animals at Beijing China. All of the experimental protocols were approved by Institutional Animal Care Unit Committee in Administration Office of Laboratory Animals at Beijing China (B10831).

### Mouse Model of Associative Memory

To analyze cell-specific mechanism for associative memory we used C57 Thy1-YFP/GAD67-GFP mice (Zhang et al., 2013) whose glutamatergic neurons were genetically labeled by yellow fluorescent protein (YFP) and GABAergic neurons were labeled by GFP.

Two groups of mice in postnatal days 20 were trained by the simultaneous pairing of mechanical whisker stimulus (WS) in the right side with odor stimulus (OS, butyl acetate toward the noses) and the unpairing of these stimulations (control), respectively (Wang et al., 2015). The paired or unpaired WS and OS were given by a multiple-sensory modal stimulator (MSMS, pattern No. 201410499466), in which the intensity, time and intervals of OS and WS were precisely set. The OS was given by switching on a butyl acetate-contained tube and generating a small liquid drop in front of the mouse noses without air pressure (video in Wang et al., 2015). The intensity of butyl acetate odor was sufficient to induce the responses of olfactory bulb neurons detected by two-photon imaging (Wang et al., 2015). The stimulated whiskers were contralateral to the barrel cortices that were studied in cell imaging and electrophysiology. The WS intensity suitably triggered whisker fluctuation after the end of stimuli (whisker-induced whisker motion (Wang et al., 2015)). Each of the mice was trained 20 s in each time, five times per day with 2 h of intervals for 15 days. During the training, each mouse was placed in a home-made cage. We paid attention to the following conditions, no stressful experimental condition and circadian disturbance to the mice that had normal whisking and symmetric whiskers (for details, see Wang et al., 2015).

The motion tracks of bilateral whisker were monitored by digital video camera (50 Hz) and were quantified in retraction duration and whisking frequency (MB-Ruler, version 5.0 by Markus Bader, MB-Softwaresolution, Germany). The responses of the bilateral whiskers to the odor-test (butyl acetate, 20 s) were measured before the training and at the end of each training day to quantify the onset time and levels of conditioned response (CR). CR-formation in mice was defined to meet the following criteria. The patterns of odorant-induced whisker motion were similar to those of whisker-induced whisker motion. Whisking frequency and whisker retraction time significantly increased, compared to control and before the training. This odorant-induced whisker motion was originally evoked by WS, in which odor signal induced a recall of whisker signal and then led to whisker motion (Wang et al., 2015).

The long whiskers (such as arcs 1–2) on the same side and rows were assigned for the mechanical stimulations and for the observations during the odor-test. This selection was based on the studies of cross-modal plasticity (Ni et al., 2010; Ye et al., 2012). We did not trim the short whiskers since whisker trimming elevated the excitability of the barrel cortex (Zhang et al., 2013).

To test CR-formation in the barrel cortex, we used an approach to silence this region by injecting 6-Cyano-7-nitroquinoxaline-2,3-(1H,4H)-dione (CNQX) and D-amino-5-phosphonovanolenic acid (D-AP5) into either side of the barrel cortices with glass pipettes (Matyas et al., 2010; O’Connor et al., 2010) to inhibit excitatory synapses (Zhang et al., 2013). If the associated signals were integrated in the barrel cortex for CR-formation, the silence of the barrel cortex should block odor-induced whisker motion. Before and after using CNQX and D-AP5, odorant-induced whisker motion and whisker-induced whisker motion were examined (Wang et al., 2015).

### Electrophysiological Recording *in Vivo*

The mice within 48 h after the completion of their behavior training were anesthetized by intraperitoneal injections of urethane (1.5 g/kg). In surgical operation, the anesthetic depth was set as lack of reflexes in pinch withdrawal and eyelid blinking. Body temperature was maintained by computer-controlled heating blanket at 37°C. The barrel cortices on both sides were localized based on the distribution of the superficial vessels (Zhao et al., 2012), mouse brain map (Paxinos and Watson, 2005) and their responses to the whisker stimulations (Wang et al., 2015). The craniotomy (2 mm in diameter) was made on the skull above the center of bilateral barrel cortices at 1 mm posterior to the bregma and 3.0–3.5 mm lateral to the midline. The anesthetic depth of the mice for electrophysiological recording *in vivo* was maintained at their moderate reflexes of pinch withdrawal and eyelid blinking, as well as their whiskers’ responses to test stimulation, i.e., the light anesthesia.

Local field potentials (LFP) were recorded in layers II–III of the bilateral barrel cortices by glass pipettes that contained standard pipette solution (150 mM NaCl, 3.5 mM KCl and 5 mM HEPES). The resistance of the recording pipettes was 5–7 MΩ. Electrical signals were inputted to an AxoClamp-2B amplifier and pClamp 10 (Axon Instrument Inc., Union City, CA, USA) for data acquisition and analysis. The electrical signals were digitized at 10 kHz and filtered by low-pass at 0.5 KHz. In data analyses, the band-pass filter (1–100 Hz) and the second order “Savitzky-Golay” filter were used to isolate LFP signals. LFP signals were complex and variable. Individual LFP events induced by WS or OS lasted for 10 ms with the sharp negative response. The differences between negative peak and baseline in individual LFPs were measured and averaged to show stimulus-evoked LFP amplitude. LFP frequency was calculated as one over inter-event intervals in 1 s, or the number of spikes in 1 s, and then averaged from the recording period. It is noteworthy that LFP recordings on both sides of the barrel cortices were done in their identical area (Zhao et al., 2012), which allowed us to compare the data about neuronal encoding in the barrel cortices.

In electrophysiological recordings, the test stimulations by odorant and whiskers’ deflection were given to the mice. The odor-test to the noses or the mechanical pulses to the whiskers on the contralateral side of the recorded barrel cortices were given to induce neuron responses, in which the parameters of stimulus intensity, frequency and duration were consistent with those in behavioral trainings. In the sequential WS and OS, inter-pulse intervals were 60 s.

### Neural Tracing and Synapse Formation

The structural connections between cortical regions were traced by injecting pAAV-SynaptoTag-mCherry-GFP (a gift from Dr. Tom Sudhof) into the trained barrel cortex and by detecting AAV-GFP presence in its CBC. The barrel cortices for AAV injection and presence detections were symmetric and posterior parts corresponding to the long whiskers that were trained by pairing WS and OS. The mice used in neural tracing were strain C57 Thy1-YFP mice whose glutamatergic neurons were genetically labeled by YFP (Zhang et al., 2013). The working principle of this AAV was that Synapsin-I promoter initiates the expression of EGFP-synaptobrevin-2 in presynaptic boutons and terminals as well as the expression of mCherry in the entire neurons, especially the axons (Xu and Südhof, 2013). In pAAV injection for one time before training the mice, glass pipettes were positioned in the barrel cortex (1 mm posterior to the bregma, 3.0 mm lateral to the midline and 0.5–1 mm in the depth), based on the map from the Mouse Brain in the Stereotaxic Coordinates (Paxinos and Watson, 2005). Three weeks after the injection into the trained barrel cortices, axon projection and synapse formation were analyzed in the contralateral side of injections in the same coronal section. CR-formation and control mice were anesthetized by the intraperitoneal injection of pentobarbital and were perfused by 4% paraformaldehyde in 0.1 M phosphate buffer solution (PBS) into left ventricle-aorta until their bodies were rigid. The brains were isolated and fixed in this solution for additional 24 h. The cortical tissues were sliced in the coronal section including the barrel cortices at 100 μm by a Vibratome. The sections were rinsed by PBS for three times, air-dried and cover-slipped for the imaging study. In order to clearly show three dimension images for new putative synapses in the barrel cortex, we placed the brain slices into Sca/eA2 solution for a few hours in order to make them transparent (Hama et al., 2011).

mCherry was used to trace axon projection. Its excitation wavelength was 561 nm and emission wavelength was 610 nm under a confocal microscopy (Nikon A1R plus, Japan). The strength of the axon innervations to the contralateral side was calculated based on the relative intensity of mCherry fluorescent in theory, i.e., fluorescent in the projection area is divided by fluorescent in the injection area. As the regions of AAV injections influenced the number of transfected neurons and the density of their axon projections, the final calculation of axon innervation strength was corrected by the injection area-size, i.e., the values of axon innervation strength were calculated by a formula that the multiplication of fluorescent intensity and mCherry area in AAV-projected locations was divided by the multiplication of fluorescent intensity and mCherry area in AAV-injected locations.

In confocal images, the contacts between GFP-labeled axon boutons and apical dendritic spines on YFP-labeled glutamatergic neurons in the barrel cortices were counted as new putative synapses. The separations of GFP-boutons and YFP-spines were done by setting the optical grating in 505–515 nm for GFP and the optical grating in 545–555 nm for YFP. The detailed information about the un-mixing of fluorescent imaging is given in Figure S1. These images were merged to construct the newly formed synapses. We defined the contacts as the putative synapses if the separation between presynaptic and postsynaptic units was less than 0.1 μm. The synaptic contacts per 100 μm dendrites were presented. Dendritic synapses in layers II–III of the barrel cortices were analyzed by using public software ImageJ (version. 1.47; National Institute of Health, Bethesda, MD, USA) and a commercialized software Imaris (version 7.2.3; Bitplane, England). In confocal imaging, the resolution was 0.05 μm per pixel, such that the minimal pixels for the measured spines and synapses were at least 9–10 in a line. To quantify the newly formed synapses, we calculated the synapse contacts between GFP-labeled presynaptic boutons and YFP-labeled postsynaptic spines per 100 μm dendrites, as well as the percentage of the dendrites that included synapse contacts. As YFP does not label all of the glutamatergic neurons due to weak Thy1 promoter, GFP-synaptobrevin2-labeled presynaptic boutons may innervate the spines on non-YFP neurons, such that the densities of GFP-labeled boutons were calculated, i.e., GFP-synaptobrevin2-labeled boutons per mm^3^.

### Brain Slices and Neurons

Cortical slices (400 μm) were prepared from the mice of CR-formation and unpaired controls. They were anesthetized by inhaling isoflurane and decapitated by a guillotine. The slices were cut by Vibratome in the oxygenated (95%O_2_/5%CO_2_) artificial cerebrospinal fluid (ACSF), in which the chemical concentrations (mM) were 124 NaCl, 3 KCl, 1.2 NaH_2_PO_4_, 26 NaHCO_3_, 0.5 CaCl_2_, 4 MgSO_4_, 10 dextrose, and 5 HEPES, pH 7.35 at 4°C. The slices were held in the oxygenated ACSF (124 NaCl, 3 KCl, 1.2 NaH_2_PO_4_, 26 NaHCO_3_, 2.4 CaCl_2_, 1.3 MgSO_4_, 10 dextrose, and 5 HEPES, pH 7.35) at 25°C for 2 h. The slices were transferred to submersion chamber (Warner RC-26G) that was perfused with the oxygenated ACSF at 31°C for whole-cell recording (Wang and Kelly, 2001).

Electrophysiological recordings on the neurons in layer II–III of the barrel cortex were conducted under DIC-fluorescent microscope (Nikon FN-E600, Japan). The wavelength at 488 nm excited GFP, and the wavelength at 575 nm excited YFP. GABAergic neurons showed basket shape and fast spiking with less adaptation in spike amplitude and frequency (Wang et al., 2008; DeFelipe et al., 2013; Lu et al., 2014). Glutamatergic neurons showed pyramidal shape and regular spikes with the adaptation of spike amplitude and frequency. The cerebral slices were coronal sections including the barrels correspondent to the projection from long whiskers that were stimulated in pairing WS and OS training.

### Whole-Cell Recording

Cortical neurons were recorded by MultiClamp-700B amplifier in voltage-clamp for their synaptic activities. Electrical signals were projected into pClamp-10 (Axon Instrument Inc., Union City, CA, USA) for data acquisition and analyses. Output bandwidth in this amplifier was 3 kHz. The pipette solution for studying excitatory synapses included (mM) 150 K-gluconate, 5 NaCl, 5 HEPES, 0.4 EGTA, 4 Mg-ATP, 0.5 Tris-GTP and 5 phosphocreatine (pH 7.35; Ge et al., 2011, 2014). The solution for studying inhibitory synapses contained (mM) 130 K-gluconate, 20 KCl, 5 NaCl, 5 HEPES, 0.5 EGTA, 4 Mg-ATP, 0.5 Tris–GTP and 5 phosphocreatine (Zhang et al., 2012). Pipette solutions were freshly made and filtered (0.1 μm), osmolarity was 295–305 mOsmol and pipette resistance was 5–6 MΩ.

Action potentials at barrel cortical neurons were induced by injecting depolarization pulses. The capability to convert excitatory inputs into digital spikes was evaluated by input-outputs (spikes vs. normalized stimuli) when the gradually increased depolarizations were given (Chen et al., 2006).

The functions of excitatory synapses were assessed based on recording spontaneous excitatory postsynaptic currents (sEPSC) at GABAergic or glutamatergic neurons while 10 μM bicuculline was added in the ACSF to block ionotropic GABA_A_ receptors (Wang, 2003). 10 μM CNQX and 40 μM D-AP5 were added into ACSF perfused to the slices at the end of experiments to test whether synaptic responses were mediated by GluR, which blocked EPSCs in our studies.

GABAergic synapses were evaluated by recording spontaneous inhibitory postsynaptic currents (sIPSC) on glutamatergic neurons in the presence of 10 μM CNQX and 40 μM DAP-5 to block ionotropic glutamatergic receptors (Zhang et al., 2012). Bicuculline (10 μM) was washed onto the slices at the end of experiments to test whether synaptic responses were mediated by GABA_A_R, which blocked sIPSC in our studies. As the pipette solution with the high concentration of chloride ions made reversal potential to be −42 mV, sIPSCs were inward when the membrane holding potential was at −65 mV (Zhang et al., 2012).

The recording of spontaneous postsynaptic currents, instead of the evoked postsynaptic current, is based on the following reasons. The amplitudes of sEPSCs and sIPSCs represent the responsiveness and the densities of postsynaptic receptors. The frequencies of sEPSCs and sIPSCs indicate the release probability of vesicle-contained transmitters from an axon terminal and the number of presynaptic inputs on each recorded neuron (Zucker and Regehr, 2002; Stevens, 2004). These parameters can be used to analyze presynaptic and postsynaptic mechanisms as well as to compare them with morphological data about the neuronal interaction, whereas the evoked postsynaptic currents cannot separate these mechanisms out. It is noteworthy that we did not use TTX into the ACSF to record miniature postsynaptic currents since we had to record neuronal spiking capability. In this regard, sEPSCs and sIPSCs recorded in our studies may include spontaneous action potential-generated and miniature synaptic events.

Data were analyzed if the recorded neurons had the resting membrane potentials negatively more than −60 mV, and action potential amplitudes more than 100 mV. The criteria for the acceptance of each experiment also included less than 5% changes in resting membrane potential, spike magnitude, and input resistance throughout each experiment. Series and input resistances in all of the neurons were monitored by injecting hyperpolarization pulses (5 mV/50 ms), and calculated by voltage pulses vs. instantaneous and steady-state currents. To estimate the effects of associative learning on neuronal spikes and synaptic transmission, we measured sEPSC, sIPSC and neuronal input-output in the slices from the mice of control and CR-formation. Their values were presented as mean ± SE. The amplitude and frequency of sEPSCs and sIPSCs were statistically compared based on the values at 67% of their cumulative probability (Wen et al., 2015; Xu et al., 2015; Ma et al., 2016).

### Statistical Analyses

The paired *t*-test was used in the comparisons of the experimental data before and after associative learning, before and after blocking synaptic transmission, the neuronal responses to WS and odorant stimulus as well as the responses in the left vs. right side in each of the mice. One-way ANOVA with *post hoc* comparisons by Student–Newman–Keuls test were used for the statistical comparisons in the changes of neuronal activity and morphological quantification between control and associative learning groups. It is noteworthy that our data has been tested to be normal distribution with equal variances before statistical comparisons.

## Results

### Neurons in Bilateral Barrel Cortices Are Recruited to Encode Odor Signal after Associative Learning

**Figure 1:**
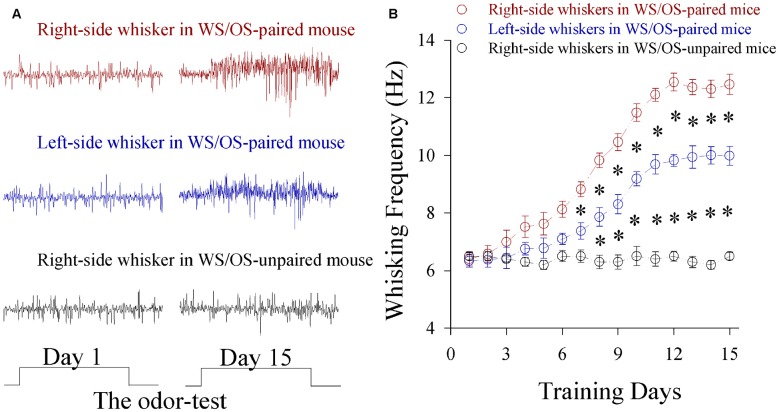
**A simultaneous pairing of unilateral whisker stimulus (WS) and olfactory stimulus (OS) induces the associated activation of the barrel and piriform cortices and leads to odorant-induced bilateral whisker motion.** The training paradigm was the pairing of WS to the right-side whiskers and OS for 15 days. The durations for OS- and WS-tests were 20 s. **(A)** Top shows whisking traces on the right-side whiskers (red) in response to the odor test before and after WS/OS-pairing. Middle shows whisking traces on the left-side whisker (blue) in response to the odor test before and after WS/OS-pairing. Bottom shows whisking traces on the right-side whiskers (black) in response to the odor test before and after WS/OS-unpairing (control). **(B)** Illustrates whisking frequency vs. training days in the right-side whiskers (red symbols) and the left whiskers (blues) from the mice of receiving WS/OS-pairing, as well as in the right-side whiskers from control mice without WS/OS-pairing, in which *p* values include *p* < 0.0001 (effect of time), *p* < 0.0001 (effect of experimental group) and *p* < 0.0001 (interaction for time and experimental group). Asterisks corresponding to each training day illustrate statistical difference *p* < 0.05 (two-way repeated measures ANOVAs with *post hoc* S-N-K test).

Mice were treated by simultaneously pairing WS in the right-side and OS for 15 days. Their whisker motions in response to the odor-test are similar to whisking induced by WS, i.e., CRs, in which the odor signal evokes whisker signal recall and whisker motion (Wang et al., 2015). Figure 1B shows bilateral whisking frequencies vs. training days. Whisking on both sides increases significantly in WS/OS-paired group (*n* = 20), compared to WS/OS-unpaired control (*n* = 20).Whisking frequencies are higher on the trained side whiskers (right-side) than on contralateral side (left-side). This dominance of odorant-induced whisker motion on the trained side may interpret that the operative capability and precision are greater in the training side than its contralateral side.

**Figure 2:**
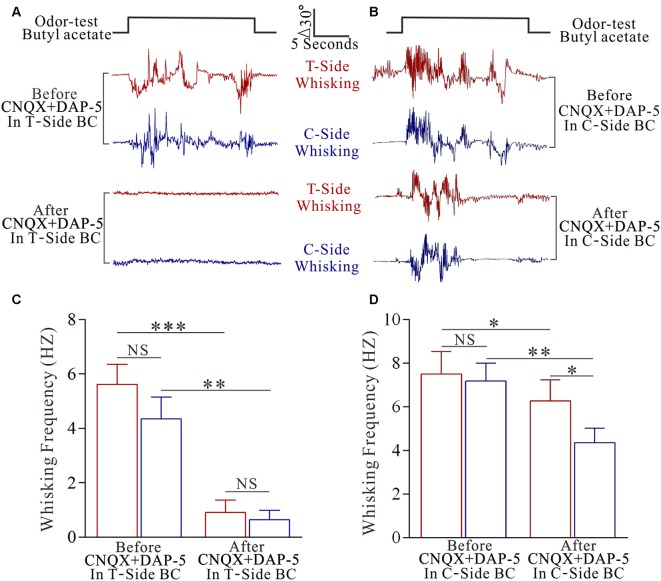
**The inhibition of pair-activated barrel cortex blocks odorant-induced bilateral whisker motion.** 10 μM 6-Cyano-7-nitroquinoxaline-2,3-(1H,4H)-dione (CNQX) and 40 μM D-amino-5-phosphonovanolenic acid (D-AP5) were injected by glass pipettes into either side of the barrel cortices. **(A)** Shows whisker traces from the training-side (T-side, red traces) and the contralateral side (C-side, blue traces) in response to the odor test (black pulse) before (middle traces) and after using CNQX and D-AP5 (bottom traces) in the T-side. The calibration bars are 30° and 5 s. **(B)** Shows whisking frequencies of T-side (red bars) and C-side (blue bars) in response to the odor test before and after using CNQX and D-AP5 in the T-side, in which *p* values include *p* < 0.0001 (effect of functional silenced), *p* = 0.2319 (effect of experimental group) and *p* = 0.4266 (interaction for functional silence and experimental group). NS shows no statistical significance. Three asterisks denote *p* < 0.001 and two asterisks denote *p* < 0.01 (two-way repeated measures ANOVA with *post hoc* S-N-K test, *p* < 0.0001). **(C)** Illustrates the whisker traces from the training-side (T-side, red traces) and the contralateral side (C-side, blue traces) in response to the odor test (black pulse) before (middle traces) and after applying CNQX and D-AP5 (bottoms) in the C-side. **(D)** Illustrates the whisking frequencies of T-side (red bars) and C-side (blue bars) in response to the odor test before and after using CNQX and D-AP5 in C-side, in which *p* values include *p* < 0.0001 (effect of functional silenced), *p* = 0.0026 (effect of experimental group) and *p* = 0.0239 (interaction for functional silence and experimental group). NS shows no statistical significance. Asterisk denotes *p* < 0.05 and two asterisks denote *p* < 0.01 (two-way repeated measures ANOVA with *post hoc* S-N-K test, *p* = 0.0011).

To make sure a primary role of the trained barrel cortices in odorant-induced motions of bilateral whiskers, we inhibited glutamatergic synaptic activities by using CNQX and DAP-5 in the trained barrel cortices at the mice that expressed odorant-induced whisker motion. This inhibition blocks odroant-induced bilateral whisker motions (Figures 2A,B), indicating the primary role of the trained barrel cortex in bilateral cross-modal responses. In addition, inhibiting the contralateral side of the trained barrel cortex partially blocks odroant-induced whisker motions in the bilateral sides (Figures 2C,D), i.e., the contralateral side of the trained barrel cortex is also involved in bilateral cross-modal responses.

**Figure 3:**
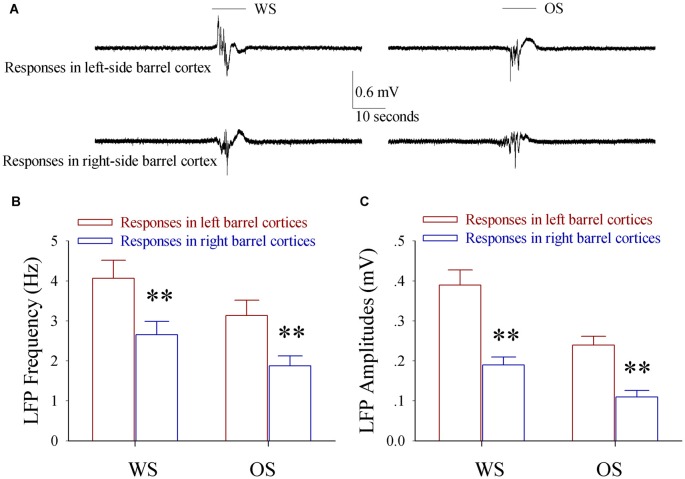
**The associated activation of the barrel and piriform cortices leads to the responses of bilateral barrel cortices to the odor and whisker signals.** Local field potentials (LFP) *in vivo* were recorded in both sides of the barrel cortices from the mice of expressing odorant-induced bilateral whisker motion. The test stimuli included butyl acetate toward their noses and the mechanical stimuli to the training-side (right side) whiskers. **(A)** Shows LFP recordings from left-side barrel cortex (top traces) and right-side barrel cortex (bottom) in responses to whisker signal (WS, left traces) and odor signal (OS, right traces). The top bars present the durations of WS (left) and OS (right). Calibration bars are 0.6 mV and 10 s. **(B)** Shows the frequencies of the* in vivo* LFPs recorded from the left-side barrel cortices (red bars, *n* = 9) and the right-side barrel cortices (blue bars, *n* = 9) in responses to WS (left bars) and OS (right bars). Two asterisks are *p* < 0.01 (paired *t*-test). **(C)** Shows the amplitudes of the* in vivo* LFPs recorded from the left-side barrel cortices (red bars, *n* = 9) and the right-side barrel cortices (blue bars, *n* = 9) in responses to WS (left bars) and OS (right bars). Two asterisks are *p* < 0.01 (paired *t*-test).

In terms of cellular mechanism for this unilateral training toward bilateral memory, we examined whether bilateral barrel cortical neurons became able to encode the newly acquired odor signal and innate whisker signal in the mice that showed odorant-induced bilateral whisker motions by recording LFP *in vivo*. To make the consistent uses of terms, we defined the right side of whiskers that received the paired WS and OS as the trained side, the left side of whiskers as the control side, the left side of barrel cortices that receive tactile signal from the trained side whiskers as the trained barrel cortices, and the right side of barrel cortices that encode tactile signal from the control side whiskers as the control barrel cortices. The neurons in both sides of the barrel cortices from a trained mouse respond to WS and OS as well as express different response patterns (Figure 3A). LFP frequencies in response to the trained side WS are 4.07 ± 0.45 Hz in the trained barrel cortices (red bar) and 3.14 ± 0.38 Hz in the control barrel cortices (blue in Figure 3B; *p* < 0.01, *n* = 9 mice). LFP frequencies in response to the OS are 2.66 ± 0.3 Hz in the trained barrel cortices (red) and 1.88 ± 0.25 Hz in the control barrel cortices (blue in Figure 3B; *p* < 0.01, *n* = 9 mice). LFP amplitudes in response to the trained side WS are 0.39 ± 0.04 mV in the trained barrel cortices (red bar) and 0.24 ± 0.02 mV in the control barrel cortices (blue in Figure 3C; *p* < 0.01, *n* = 9 mice). LFP amplitudes in response to the OS are 0.19 ± 0.02 mV in the trained barrel cortices (red) and 0.11 ± 0.02 mV in the control barrel cortices (blue in Figure 3B; *p* < 0.01, *n* = 9 mice). Moreover, LFPs in response to WS and OS at the same-side barrel cortices are statistically different (*p* < 0.01). On the other hand, the neurons in both-side barrel cortices from WS/OS-unpaired mice (control) respond to their correspondent WS, but not respond to the OS and the control-side WS (Figure S2). Thus, bilateral barrel cortical neurons are recruited to encode the newly learnt odor signal and innate whisker signal after pairing unilateral WS and OS. These associative memory cells may work for unilateral training toward bilateral memory. The different patterns in response to the WS and OS indicate their abilities to distinguish these associated signals during information retrieval (Wang et al., 2015).

The recruitment of associative memory neurons in bilateral barrel cortices for unilateral training toward bilateral memory is hypothetically driven by the formation of new synapse innervations between bilateral barrel cortices.

### Synaptic Connections Between Bilateral Barrel Cortices Are Established after Associative Learning

**Figure 4:**
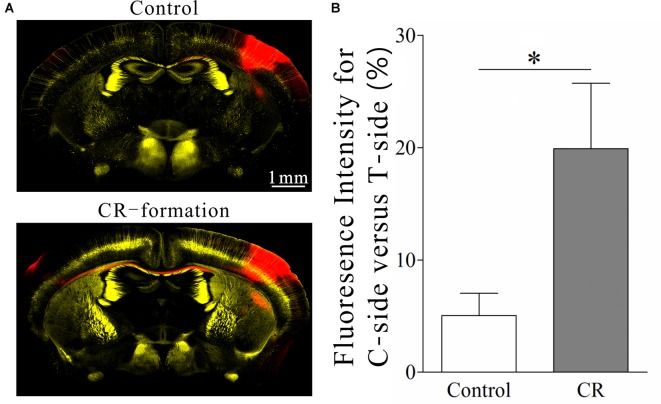
**A co-activation of the barrel and piriform cortices leads to the axon projection from the neurons in the activated barrel cortex toward the ipsilateral piriform cortex (IPC) and strengthens the axon projections to the contralateral barrel cortex (CBC).** Neural tracing was done by injecting pAAV-SynaptoTag-mCherry-green fluorescent protein (GFP) into left-side barrel cortex (the contralateral side of the trained whiskers) and detected by scanning mCherry red for the axons and GFP for the presynaptic boutons. Glutamatergic neurons in mouse cerebral cortices were genetically labeled by yellow fluorescent protein (YFP; strain C57 by Thy1 promoter). **(A)** Top panel illustrates an imaging of coronal brain section from a control mouse. After the injection of AAV into the left-side barrel cortex for 3 weeks, the mCherry red is barely detected in its CBC. Bottom panel illustrates an imaging of coronal brain section from a mouse of expressing odorant-induced bilateral whisker motion (conditioning response, CR). After the injection of AAV into the activated barrel cortex for 3 weeks, the mCherry red is detected in the IPC and its CBC. **(B)** Demonstrates the ratios of fluorescent intensity in the contralateral side (right-side) to that in the activated side (left-side) from the mice of controls (white bar) and of CR-formation (gray bar). One asterisk is *p* < 0.05 (unpaired *t*-test).

**Figure 5:**
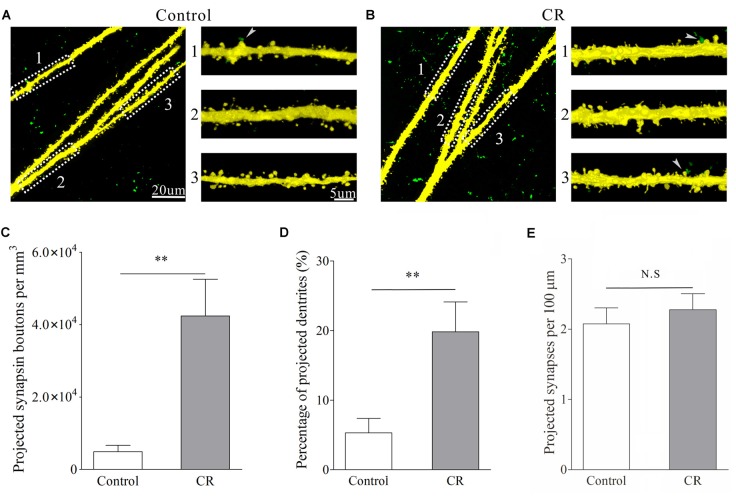
**An associated activation of the barrel and piriform cortices leads to the axon projection from the pair-activated barrel cortex toward its CBC to make new putative synapses.** Glutamatergic neurons in the mouse cerebral cortices were genetically labeled by YFP. Synapse formation was traced by injecting pAAV-SynaptoTag-GFP into the pair-activated barrel cortex and by detecting the GFP-labeled presynaptic terminals as well as the contacts between GFP-labeled presynaptic boutons and YFP-labeled postsynaptic spines in the CBC. **(A)** Shows the images from control mouse. After the injection of AAV into the pair-activated barrel cortex for 3 weeks, presynaptic boutons (green dots) and synaptic contacts (green-yellow dots pointed by white arrows) are barely detected in CBC. **(B)** Illustrates the images from a CR-formation mouse. After the injection of AAV into the pair-activated barrel cortex for 3 weeks, the presynaptic boutons and synapse contacts are obviously detected in its CBC. **(C)** Shows presynaptic boutons per mm^3^ in the contralateral side of the activated barrel cortex from control mice (white bar) and CR-formation (gray). Two asterisks are *p* < 0.01 (unpaired *t*-test). **(D)** Shows the percentage of apical dendrites that receive presynaptic boutons to form synapse contacts vs. total dendrites in the contralateral side of the pair-activated barrel cortex from control mice (white) and CR-formation (gray). Two asterisks are *p* < 0.01 (unpaired *t*-test). **(E)** Shows synapse contacts per 100 μm of dendrite in the contralateral side of the pair-activated barrel cortex from control mice (white bar) and CR-formation (gray).

Synaptic connections were traced by injecting pAAV-SynaptoTag-mCherry-GFP into the trained barrel cortex and detecting its axon projections on contralateral side, where cortical glutamatergic neurons were genetically labeled by YFP (Zhang et al., 2013). The relative intensity of mCherry fluorescent was calculated to indicate axon projections. The densities of green presynaptic boutons as well as the contacts formed between GFP-labeled boutons and YFP-labeled spines were used to merit the newly formed synapses (see “Materials and Methods” Section). Compared with control mice, mCherry is increasingly detected in the contralateral sides of the trained barrel cortices from the mice that show bilateral cross-modal responses (Figures 4A,B, *p* < 0.01, *n* = 10 mice). In addition, presynaptic boutons (GFP-labeled boutons per mm^3^ in Figures 5A–C) and new putative synapses (the percentage of dendrites with the contacts and the contacts per 100 μm dendrite in Figures 5A,B,D,E) are highly detected in the mice that express bilateral cross-modal responses vs. controls (*p* < 0.01, *n* = 10 mice and *n* = 100 apical dendrites for each group). These results indicate the significant addition of new putative synapses in the contralateral barrel cortices that are innervated by the axons from the trained barrel cortices after the associaitve bilateral memory is established. It is noteworthy that axon projections from the non-trained barrel cortex to its contralateral side not changed in CR-formation mice vs. controls (Figure S3).

These axon projections and new putative synapses from the trained barrel cortex to its CBC may drive the recruitment of associative memory cells, which may contribute to unilateral training toward bilateral memory (Figures 1–3).

### Glutamatergic Neurons in Bilateral Barrel Cortices Are Differentially Upregulated

In addition to axon innervation and synapse formation, the recruitments of glutamatergic neurons to be associative memory cells for bilateral memory may be caused by the upregulation of their activities and the downregulation of their inhibitory synapses. We examined this hypothesis by recording YFP-labeled glutamatergic neurons in bilateral barrel cortices from the mice that expressed odorant-induced bilateral whisker motions vs. the controls. sEPSC were recorded to evaluate glutamatergic synapse efficacy, input-outputs were analyzed to assess neuronal intrinsic property and sIPSC were recorded to evaluate GABAergic synaptic transmission (Zhang et al., 2013). The statistical comparisons for the amplitude and frequency of sEPSCs and sIPSCs were conducted based on the values at 67% of their cumulative probability (Wen et al., 2015; Xu et al., 2015; Ma et al., 2016).

**Figure 6:**
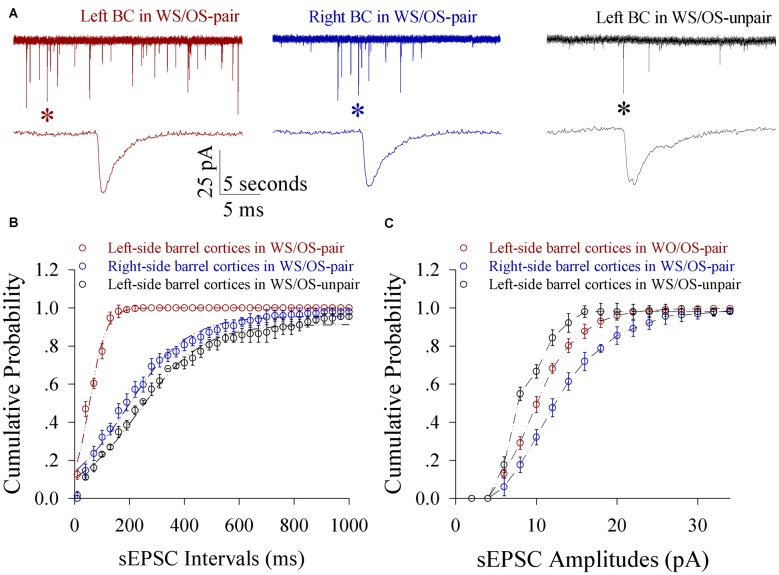
**Excitatory synaptic transmission on glutamatergic neurons from the pair-activated barrel cortex and its CBC rises in the mice that show expressing odorant-induced bilateral whisker motion (CR-formation).** Spontaneous excitatory postsynaptic currents (sEPSC) were recorded on barrel cortical glutamatergic neurons in brain slices under voltage-clamp (holding potential, −70 mV) in the presence of 10 μM bicuculline. **(A)** Illustrates sEPSCs recorded on a neuron of the left barrel cortex (the pair-activated side, red trace) and the right barrel cortex (the contralateral side, blue) from a CR-formation mouse, and the left barrel cortex from a control (dark), respectively. Bottom traces are the expanded waveforms that are selected from top traces and marked by asterisks. Calibration bars are 25 pA, 5 s (top) and 5 ms (bottom). **(B)** Illustrates cumulative probability vs. inter-sEPSC intervals from the neurons in left barrel cortices of controls (dark symbols, *n* = 15 neurons from nine mice), in the left barrel cortices (reds, *n* = 15 neurons from nine mice) and in the right barrel cortices (blues, *n* = 15 neurons from nine mice) from CR-formation mice. **(C)** Illustrates cumulative probability vs. sEPSC amplitudes from the neurons in left barrel cortices of controls (dark symbols, *n* = 15 neurons from nine mice), in the left barrel cortices (reds, *n* = 15 neurons from nine mice) and in the right barrel cortices (blues, *n* = 15 neurons from nine mice) from CR-formation mice.

Excitatory synaptic transmission on glutamatergic neurons increases in both-side barrel cortices from CR-formation mice. sEPSC amplitude and frequency appear higher in CR-formation mice than controls (Figure 6A). Figure 6B shows cumulative probability vs. inter-sEPSC intervals in the neurons of pair-trained barrel cortices from CR-formation mice (red symbol, *n* = 15), of control barrel cortices from CR-formation mice (blue, *n* = 15) and of unpair-trained barrel cortices from control mice (black, *n* = 15). Statistical analysis illustrates that sEPSC frequencies (1/inter-EPSC intervals) are higher in pair-trained barrel cortices from CR-formation mice than in control barrel cortices from CR-formation mice and unpair-trained barrel cortices from control mice (*p* < 0.01). Figure 6C shows cumulative probability vs. sEPSC amplitudes in the neurons of pair-trained barrel cortices from CR-formation mice (red symbol, *n* = 15), of control barrel cortices from CR-formation mice (blue, *n* = 15) and of unpair-trained barrel cortices from control mice (black, *n* = 15). sEPSC amplitudes show the high to low grade in control barrel cortices from CR-formation mice, pair-trained barrel cortices from CR-formation mice and unpair-trained barrel cortices from control mice (*p* < 0.01). Associative learning enhances excitatory synaptic function in barrel cortical glutamatergic neurons.

**Figure 7:**
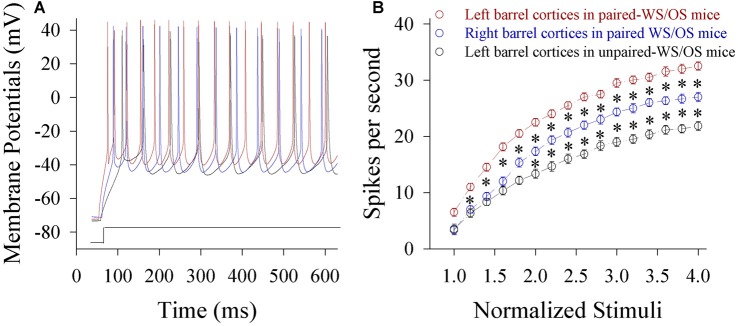
**The ability to encode spikes on glutamatergic neurons from the pair-activated barrel cortex and its CBC rises in the mice that show odorant-induced bilateral whisker motion (CR-formation).** The sequential spikes were induced by depolarization pulses under current-clamp recordings on barrel cortical glutamatergic neurons in the brain slices. **(A)** Traces show the spikes on the neurons by the depolarization pulse with same intensity in left barrel cortex from a CR-formation mouse (red), right barrel cortex from a CR-formation mouse (blue) and left barrel cortex from a control mouse (black). **(B)** Shows spikes vs. normalized stimuli from the neurons in left barrel cortices from control mice (dark symbols, *n* = 15 neurons from nine mice) as well as in left barrel cortices (reds, *n* = 15 neurons from nine mice) and right barrel cortices (blues, *n* = 15 neurons from nine mice) from CR-formation mice, in which *p* values include *p* < 0.0001 (effect of stimulus intensity), *p* < 0.0001 (effect of experiment group) and *p* < 0.0001 (interaction for stimulus intensity and experiment group). Asterisks corresponding to each stimulus intensity show statistical difference *p* < 0.01 (two-way repeated measures ANOVAs with *post hoc* S-N-K test).

Spiking capability at glutamatergic neurons increases in both-side barrel cortices from CR-formation mice. Figure 7A shows spiking abilities at glutamatergic neurons in the pair-trained barrel cortex from a CR-formation mouse (red trace), the control barrel cortex from a CR-formation mouse (blue) and the unpair-trained barrel cortex from a control mouse (black). Figure 7B shows input-output curves of glutamatergic neurons in pair-trained barrel cortices from CR-formation mice (red symbol, *n* = 15), control barrel cortices from CR-formation mice (blue, *n* = 15) and unpair-trained barrel cortices from control mice (black, *n* = 15) Statistical analysis indicates that spiking abilities from high to low grade are in pair-trained barrel cortices from CR-formation mice, control barrel cortices from CR-formation mice and unpair-trained barrel cortices from control mice, respectively (*p* < 0.01). Associative learning enhances the capability to convert excitatory inputs into digital spikes in barrel cortical glutamatergic neurons.

**Figure 8:**
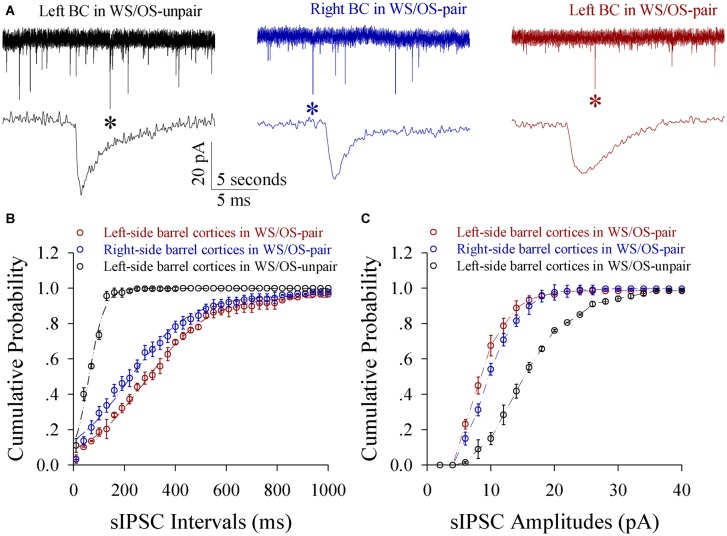
**Inhibitory synaptic transmission on glutamatergic neurons from the pair-activated barrel cortex and CBC decreases in the mice that show odorant-induced bilateral whisker motion (CR-formation).** Spontaneous inhibitory postsynaptic currents (sIPSC) were recorded on barrel cortical glutamatergic neurons in brain slices under voltage-clamp (holding potential, −70 mV) in the presence of 10 μM CNQX and 40 μM D-AP5. **(A)** Shows sIPSCs recorded on a neuron of the left barrel cortex from a control mouse (dark trace), the right barrel cortex (the contralateral side, blue) and the left barrel cortex (the pair-activated side, red) from a CR-formation mouse, and, respectively. Bottom traces are expanded waveforms that are selected from top traces and marked by asterisks. The calibration bars are 20 pA, 5 s (top) and 5 ms (bottom). **(B)** Illustrates cumulative probability vs. inter-sIPSC intervals from the neurons in the left barrel cortices of controls (dark symbols, *n* = 15 neurons from nine mice), in the left barrel cortices (reds, *n* = 15 neurons from nine mice) and in the right barrel cortices (blues, *n* = 15 neurons from nine mice) from CR-formation mice. **(C)** Shows cumulative probability vs. sIPSC amplitudes from the neurons in left barrel cortices of controls (dark symbols, *n* = 15 neurons from nine mice), in the left barrel cortices (reds, *n* = 15 neurons from nine mice) and in the right barrel cortices (blues, *n* = 15 neurons from nine mice) from CR-formation mice.

Inhibitory synaptic transmission on glutamatergic neurons decreases in both-side barrel cortices from CR-formation mice. The amplitudes and frequencies of sIPSCs appear lower in CR-formation mice than control mice (Figure 8A). Figure 8B illustrates cumulative probability vs. inter-sIPSC intervals in the neurons of pair-trained barrel cortices from CR-formation mice (red symbols, *n* = 15), of control barrel cortices from CR-formation mice (blue, *n* = 15) and of unpair-trained barrel cortices from control mice (black, *n* = 15). Figure 8C shows cumulative probability vs. sIPSC amplitudes in the neurons of pair-trained barrel cortices from CR-formation mice (red symbols, *n* = 15), of control barrel cortices from CR-formation mice (blue, *n* = 15) and of unpair-trained barrel cortices from control mice (black, *n* = 15). sIPSC amplitudes and frequencies (1/inter-sIPSC intervals) are significantly lower in pair-trained barrel cortices from CR-formation mice and control barrel cortices from CR-formations than unpair-trained barrel cortices from control mice (*p* < 0.01). Consistent with the attenuation of GABAergic synaptic outputs, spiking ability at GABAergic neurons decreases (Figure S4). These results appear to support the hypothesis above.

## Discussion

The associated stimulations of unilateral whiskers and olfaction lead to odorant-induced whisker motions in both sides, dominantly in the training side (Figure 1). In the mice that express this bilateral cross-modal memory, the bilateral barrel cortical neurons are recruited to encode the acquired odor signal alongside the innate whisker signal (Figure 3). New axon projections and synapse innervations are established from the pair-trained barrel cortex to its CBC (Figures 4, 5). In pair-trained barrel cortices and their contralateral side, excitability and excitatory synaptic transmission in glutamatergic neurons are upregulated (Figures 6, 7), as well as excitability and inhibitory synaptic outputs in GABAergic neurons are downregulated (Figure 8 and Figure S4). The new synapse innervations and the coordinated neuron refinement (the upregulated excitatory neuron function and the downregulated inhibitory neuron function) may drive these neurons be recruited as associative memory cells in bilateral barrel cortices for unilateral learning toward bilateral memory. Dominant synapse formation and associative memory cell recruitment in the trained barrel cortex may interpret the preferential expression of associative memory on the training-side that has more capability and precision in signal retrieval vs. on the contralateral side (Figure 1).

After sensory signals and operative skills are trained in the unilateral limbs, these signals and skills can be retrieved in bilateral limbs. The physiological impact of unilateral training toward bilateral memory is for bilateral limbs to be coordination and mutual substitute. As the corpus callosum connects two cerebral hemispheres (Witelson, 1985; Dubb et al., 2003) and coordinates the bimanual motions (Lum et al., 2011; Kozlovskiy et al., 2012; Gooijers and Swinnen, 2014), it may be involved in unilateral training toward bilateral memory. However, this structure mainly coordinates bilateral events related to motions and special sensations (Lum et al., 2011; Gooijers and Swinnen, 2014), but not those related to somatic sensation (Armstrong-James and George, 1988; Kawaguchi, 1992; Shuler et al., 2001; Glazewski et al., 2007). The afferents of special sensations, such as visual and auditory, ascend anatomically via ipsilateral and contralateral pathways for animals to detect the sources of remote signals in stereotype manner and to have predictable responses. On the other hand, somatic sensations are precise in one side of the body via their crossed afferent pathways to the contralateral side. The corpus callosum should not be involved in encoding these somatic sensations to prevent the losses of their unilateral somatosenory precision and their ability to escape away from the side of harmfulness-stimulated limbs toward the contralateral side. The upregulation of the corpus callosum (Pietrasanta et al., 2012; Steele et al., 2013) may strenghten the association of bilateral somatosensory cortices for the signal exchanges between two sides of the limbs physiologically, such as unilateral training toward bilateral memory. However, if the upregulation of callosum connectivity, such as electrical shock to feet in fear conditioning, is strong to reach a pathological threshold, it may lead to the sensitization across body-side, which remains to be examined.

**Figure 9:**
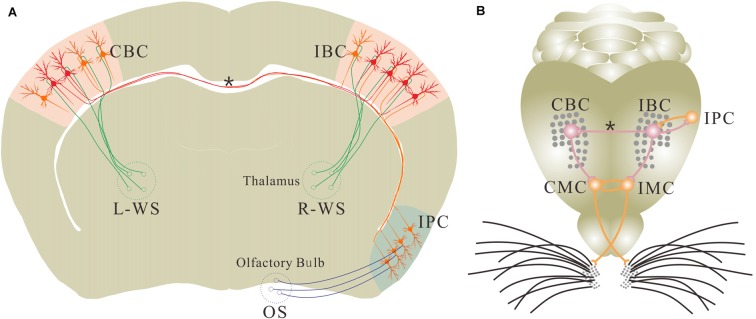
**Associative learning leads to the new axon projection and synapse formation. (A)** The associated activations of the unilateral barrel and piriform cortices induces the mutual synaptic innervations between the IPC and the ipsilateral barrel cortex (IBC) as well as the axon projection and synapse formation from this IBC to its CBC for the recruitment of associative memory cells in these cortices. These associative memory cells encode both whisker and odor signals. However, the barrel cortical neurons receive innate WS from the thalamus and the piriform cortical neurons receive innate odor signal (OS) from olfactory bulb under the normal condition. **(B)** Shows mutual synaptic innervations between the IPC and IBC as well as from this IBC to its CBC. Odorant-induced whisker motion in ipsilateral side is fulfilled by a process that the odor test activates the IPC, the IBC and the ipsilateral motor cortex (IMC). Odorant-induced whisker motion in contralateral side is fulfilled by the process that the odor test activates the IPC, the IBC, the CBC and the contralateral motor cortex (CMC). In this regard, the sensory cortices store signals and the motor cortex present signals during information retrieval. This diagram illustrates a proposed cellular mechanism underlying unilateral training toward bilateral memory. Red shows new axons, synapses and associative memory cells. Oranges shows innate neurons. Asterisk shows new axon and synapse innervation. Green neurons and axons are located in the thalamus, which carry whisker signal to the barrel cortex. OS is olfactory signal, R-WS is whisker signal from right side, and L-WS is whisker signal from left side.

This hypothesis is implied in our study. In CR-formation mice, the synaptic connections between both-side barrel cortices are upregulated (Figures 4, 5) for this unilateral training toward bilateral memory (Figure 2). In the trained barrel cortices and their contralateral sides, excitability and excitatory synaptic transmission in glutamatergic neurons are upregulated (Figures 6, 7), as well as excitability and inhibitory synaptic outputs in GABAergic neurons are downregulated (Figure 8 and Figure S4). Their coordinated changes drive the barrel cortex to the optimal excitatory state, which facilitates the recruitment of the cortical neurons to be associative memory cells that encode the newly acquired odor signal and the innate whisker signal (Figure 3). Therefore, the co-activations of barrel and piriform cortices may lead to their mutual innervations (Wang et al., 2014, 2016). The axon and synapse innervations from the piriform cortex onto barrel cortical neurons dive them to be recruited as associative memory cells, and in turn the axons of these associative memory neurons in this pair-trained barrel cortex project toward its CBC to form new synapses for the recruitment and refinement of contralateral neurons, leading to unilateral training toward bilateral memory (Figure 9). In this regard, the associative activation of the piriform and barrel cortices is the primary driving force for unilateral training toward bilateral memory. Molecular mechanism for these processes on both sides remains to be studied, in which microRNA-324 and microRNA-133a appear involved (Wang et al., 2016).

The natural whisking is usually symmetric, which is coordinated by the connections between both sides of the movement-related brain areas (Alloway et al., 2009; Pashaie and Falk, 2013; Takatoh et al., 2013), e.g., the motor cortex that receives the inputs from the barrel cortex (Alloway et al., 2004). Different from this natural whisking, the odor test induces bilateral whisker motions with higher strength in the trained side than contralateral side in the mice with unilateral training toward bilateral memory. After this bilateral memory expresses, functional synapse connections are formed from the trained barrel cortex to its contralateral side. These axon projections and synapse formations are required for odorant-induced motions in bilateral whiskers (Figure 2). In other words, this unilateral training toward bilateral memory is fulfilled by a coordination of the somatosensory cortices through their connections. Moreover, the dominant upregulation of glutamatergic synaptic transmission and neuronal excitability (Figures 6, 7) as well as the dominant downregulation of GABAergic synaptic transmission and neuronal activity (Figure 8 and Figure S4) in the trained barrel cortex, in comparison with its CBC, may also interpret the preferential expression of associative memory in the training-side that has more capability and precision during information retrieval vs. in the contralateral side (Figure 1).

It has been described that there may be low-dense connection between the bilateral barrel cortices (Shuler et al., 2001; Glazewski et al., 2007; Aronoff et al., 2010), which is detected by neural tracing in control mice (Figure 4). The function of this connection remains unknown. When one-side barrel cortex is activated by WS, this connection is insufficient to activate the neurons of its CBC in control mice (Figure S2). This connection is also insufficient to trigger onset of odorant-induced bilateral whisker motions. After the associated activations of barrel and piriform cortices, the neurons in the trained barrel cortex mutually innervate with the ipsilateral piriform cortex (IPC; Wang et al., 2014, 2015). In the meantime, these recruited neurons in the trained barrel cortex project their axons to its CBC, such that the odor signal is able to induce the responses of contralateral barrel cortical neurons. As the inhibition of co-activated barrel cortex removes odorant-induced bilateral whisker motions (Figure 2), the odor signal propagates from the piriform cortex to the barrel cortex, from where the odor signal goes toward CBC by axon projection to fulfill bilateral memory.

The bilateral retrieval of the learnt information has been well known in verbal memory, working memory and perceptual memory for special sensations, in which the corpus callosum plays an important role (Hasegawa et al., 1999; Wong, 2000; Peltier et al., 2012; Pietrasanta et al., 2012; Treble et al., 2013; Erickson et al., 2014). The two issues need to be cleared for these reports. These learning and cognitive processes are fulfilled by the special sensations and skill operations, in which the special sensory signals naturally ascend to bilateral sensory cortices through ipsilateral and contralateral afferent pathways as well as the corpus callosum is involved. However, the bilateral natural links are not sufficient for memory retrieval related to the somatic sensation (Figure 1 and Figure S2). The second, the indications from these previous studies are achieved from the splitting of two cerebral hemispheres by the surgical separations of the corpus callosum or after its traumatic injury. However, the surgical separation of bilateral specific and symmetric regions is difficult. In our study, the pharmacological silence of the unilateral barrel cortex is used by the local microinjection of glutamatergic receptor-channel antagonists (Figure 2), which prevents the extensive injury of the corpus callosum.

Together in these data, our study reveals the formation of associative memory by the recruitment of new synapse innervations and associative memory cells in the relevant cortices. Associative memory cells presumably have the following characteristics. They encode the associated signals. They receive synapse innervations from the cortices that primarily encode these associated signals. Their axons project toward motor-related cortices to initiate memory presentation. Their recruitment is regulated by the genes and proteins related to associative memory (Wang et al., 2016). The working principle of these associative memory cells may be based on the facilitation of their excitatory states driven by the newly innervated synapses from other sensory cortices. For instance, in addition to receiving whisker signal and inducing whisker motions (Figure 9), the barrel cortical neurons receive the synapse innervations from the piriform cortex. Synapse activities induced by odor signal drive these barrel cortical neurons toward the threshold to fire spikes. Their spikes in turn activate motor cortical neurons for whisker motion in the mice, i.e., odorant-induced whisker motion.

The frequencies of spontaneous synaptic activities presumably indicate the release probability of vesicle-contained transmitters from an axonal terminal and the density of presynaptic axons innervated on the recorded neuron (Zucker and Regehr, 2002; Stevens, 2004). Our results show that sEPSC frequency rises in barrel cortical neurons and new putative synapses form on barrel cortical neurons. The consistent data strengthen the reliability of our study. On the other hand, the increase of sEPSC amplitudes, which indicate receptor responses and numbers, is less in the trained barrel cortices than control barrel cortices from CR-formation mice. Although we do not know the reasons for the alterations in sEPSC amplitudes vs. frequencies, the homeostasis between transmitter release and receptor responses (Chen et al., 2008) may occur in glutamatergic synapses after associative learning. In addition, by using mice whose cortical glutamatergic and GABAergic neurons are labeled by different fluorescent proteins, we are able to clearly analyze the structural and functional refinements in cell-specific manner. By tracing the axons with AAV-synaptobrevin2-GFP and their termination onto YFP-neuronal spines, we are able to observe axon projections and synapse formations between both sides of the barrel cortices. The combined technical advances allow to elucidate neuron connectomics and synapse formations involved in unilateral training toward bilateral memory.

## Author Contributions

ZG, LC, RF, WL, DW, LH, SZ, SG and YZ contributed to experiments and data analyses. J-HW contributed to the project design and writing of the article. All authors have read and approved the final version of the manuscript.

## Conflict of Interest Statement

The authors declare that the research was conducted in the absence of any commercial or financial relationships that could be construed as a potential conflict of interest.
