# Supplementary material for: Associations of Unilateral Whisker and Olfactory Signals Induce Synapse Formation and Memory Cell Recruitment in Bilateral Barrel Cortices: Cellular Mechanism for Unilateral Training Toward Bilateral Memory
Source: Front Cell Neurosci. 2016 Dec 16;10:285. doi: 10.3389/fncel.2016.00285 (PMC5160353; doi:10.3389/fncel.2016.00285)
Supplement: Supplementary file 1 [file DataSheet_1.doc]

**Revision to: Frontiers in Cellular Neuroscience**

**Associations of unilateral whisker and olfactory signals induce synapse formation and memory cell recruitment in bilateral barrel cortices: cellular mechanism for unilateral training toward bilateral memory**

**Zilong Gao^1,2#^, Lei Chen^3#^, Ruicheng Fan^3#^, Wei Lu^4^, Dangui Wang^1^, Li Huang^3^, Shidi Zhao^3^, Sudong Guan^3^, Yan Zhu^3^ and Jin-Hui Wang^1,2,3^***

***1) State Key Lab of Brain and Cognitive Sciences, Institute of Biophysics, Chinese Academy of Sciences, Beijing China 100101; 2) College of Life Sciences, University of Chinese Academy of Sciences, Beijing China 100101; 3)Department of Pathophysiology, Bengbu Medical College, Bengbu Anhui China 233000; 4) Qingdao University, Medical College, 38 Dengzhou, Shandong China 266021***

**Running title:** Neural basis for unilateral learning to bilateral memory

**Key words:** learning, memory, glutamate, GABA, neuron, synapse, barrel cortex, whisker, olfaction

**Word counts:** abstract, 188; text, 7399

**Competing interest:** authors declare no competing interest

**#:** Gao Z, L Chen and Fan R contributed to this work equally

**Corresponding author:**

Jin-Hui Wang, Ph.D. & MD

The Institute of Biophysics, Chinese Academy of Sciences

15 Datun Road, Chaoyang District

Beijing China 100101

[jhw@sun5.ibp.ac.cn](mailto:jhw@sun5.ibp.ac.cn); 86-10-64888472

## Figure S1 The barrel cortex responds to contralateral whisker signal, but not ipsilateral whisker signal and odor signal in control mice. Local field potentials (LFP) *in vivo* were recorded in both sides of the barrel cortices from the control mice. The test stimulations included butyl acetate toward their noses and the mechanical stimuli to either the training-side (right side) whiskers or the non-training side (left side) whiskers. A) Top traces show LFP recordings from the left-side barrel cortex in responses to right-side whisker signal (R-WS, left trace), left-side whisker signal (L-WS, middle trace) and odorant signal (OS, right trace). Bottom traces show LFP recordings from the right-side barrel cortex in responses to left-side whisker signal (L-WS, left trace), right-side whisker signal (R-WS, middle trace) and odorant signal (OS, right trace). The top bars present the durations of WS and OS. Calibration bars are 0.6 mV and 10 seconds. B) shows the frequencies of the *in vivo* LFPs recorded from the left-side barrel cortices (red bars, n=9) and the right-side barrel cortices (blue bars, n=9) in responses to WS in the contralateral side (WSc in left bars), WS in ipsilateral side (WSi in middle bars) and OS (right bars). Two asterisks are p<0.01 (paired t-test). C) shows the amplitudes of the *in vivo* LFPs recorded from the left-side barrel cortices (red bars, n=9) and the right-side barrel cortices (blue bars, n=9) in responses to WS in the contralateral side (WSc in left bars), WS in ipsilateral side (WSi in middle bars) and OS (right bars). NS is no statistical change.

**
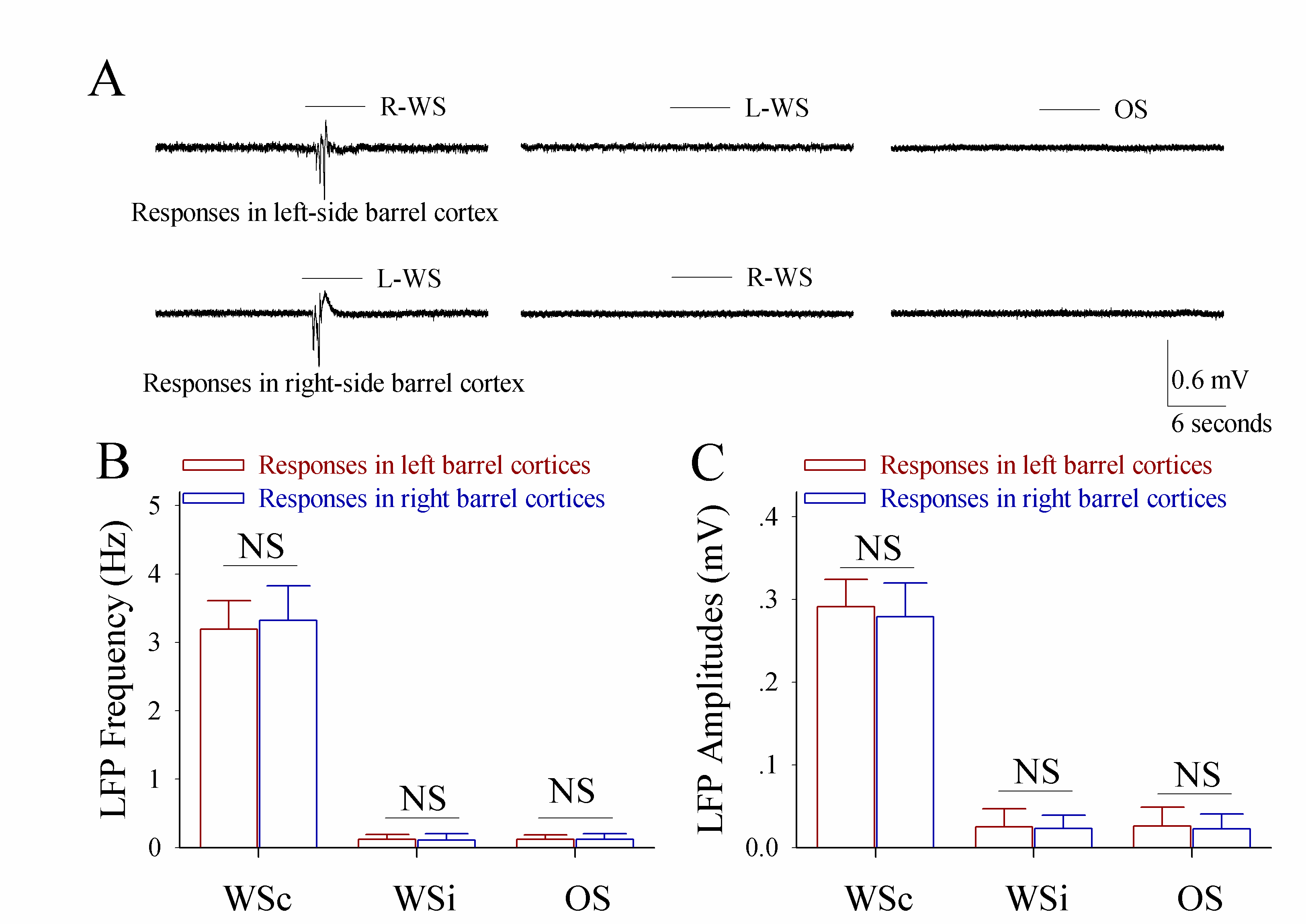
**

**Figure S2** The ability to encode spikes on GABAergic neurons from the co-activated barrel cortex and its contralateral barrel cortex decreases in the mice of expressing odorant-induced bilateral whisker motion (CR-formation). Sequential spikes were induced by depolarization pulses under current-clamp recordings on barrel cortical GABAergic neurons in the brain slices. **A)** Traces show depolarization-induced spikes on a neuron of the left barrel cortex from a CR-formation mouse. **B)** Traces show depolarization-induced spikes on a neuron of the right barrel cortex from a CR-formation mouse. **C)** Traces show depolarization-induced spikes on a neuron of the left barrel cortex from a control. **D)** illustrates spikes versus normalized stimuli from the neurons in left barrel cortices of controls (dark symbols, n=15 neurons from nine mice), in the left barrel cortices (reds, n=15 neurons from nine mice) and in the right barrel cortices (blues, n=15 neurons from nine mice) from CR-formation mice.

**
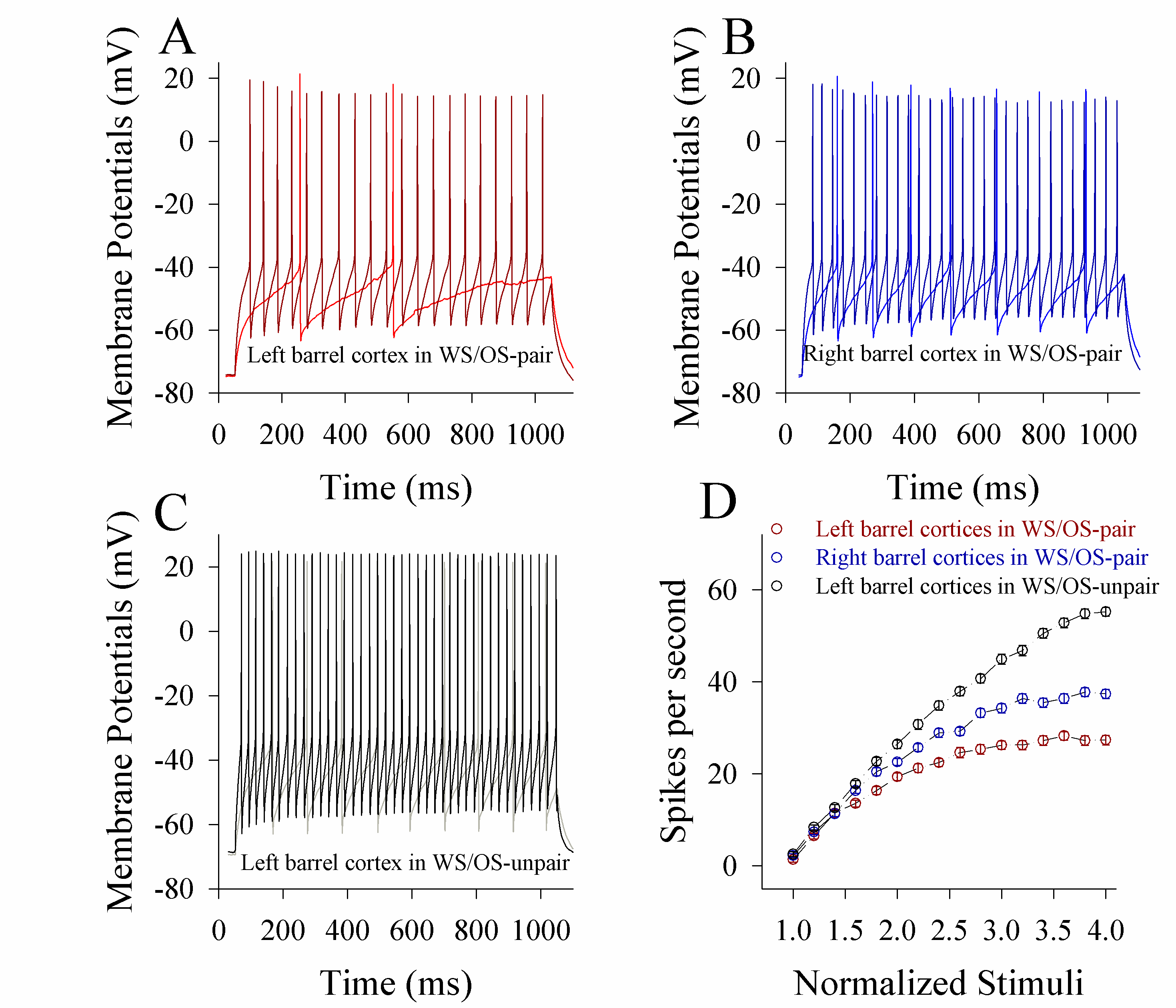
**


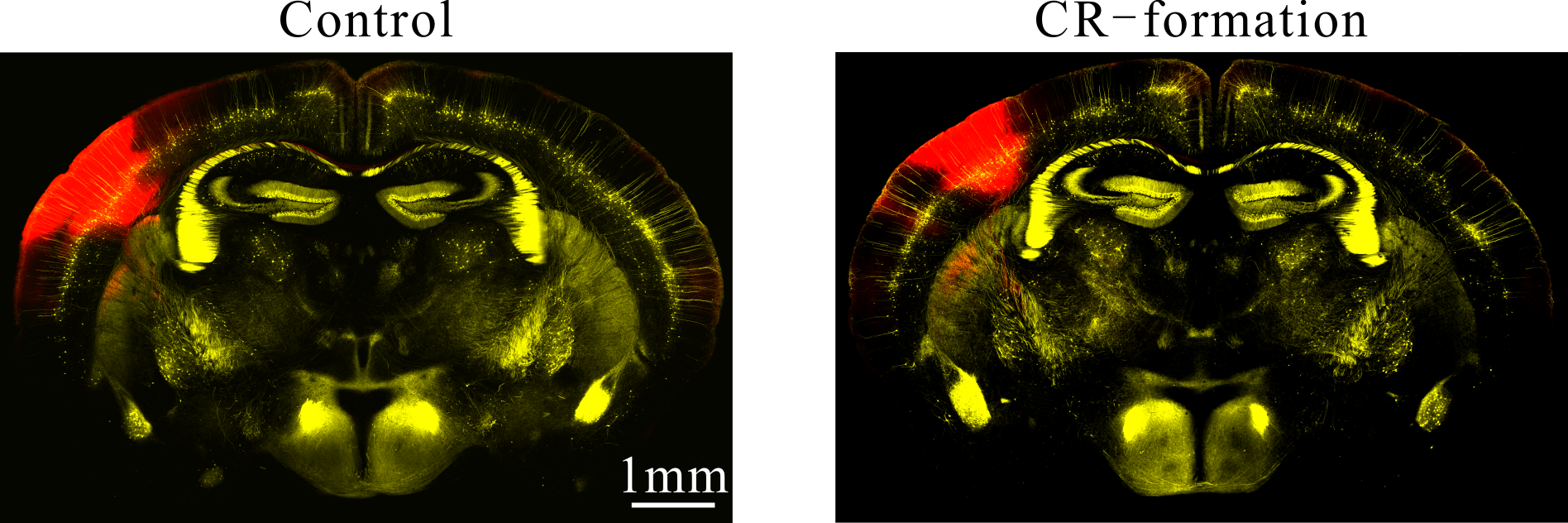
**Figure S3** The axon projections between the non-activated barrel cortex and the piriform cortex as well as the non-activated barrel cortex toward its contralateral barrel cortex are not altered in CR-formation mice and controls. Neural tracing was conducted by injecting AAV-SynaptoTag-Cherry-GFP into the right-side barrel cortex (the ipsilateral side of the trained whiskers) and detected by scanning mCherry red for the axons. Glutamatergic neurons in mouse cerebral cortices were genetically labeled by YFP (strain C57 by Thy1 promoter). Left panel illustrates an imaging of coronal brain section from a control mouse. After the injection of AAV into the right-side barrel cortex for three weeks, mCherry red is barely detected in the ipsilateral piriform cortex and its contralateral barrel cortex. Right panel illustrates an imaging of coronal brain section from a CR-formation mouse. After the injection of AAV into the right-side barrel cortex for three weeks, mCherry red is barely detected in the ipsilateral piriform cortex and its contralateral barrel cortex.

**Figure S4** Two optical gratings are used for the un-mixing of GFP and YFP. **A)** illustrates the optical spectra of GFP and YFP as well as the setting of optical gratings, in which GFP and YFP are excited by laser beam 488nm (blue). Two optical gratings are set in a range of 505~515 nm as channel one (green) and in a range of 545~555nm as channel two (red). **B)** shows the un-mixing of GFP and YFP. Left panel shows the images of dendrites from channel one, channel two and their merging in control, respectively. Right panel shows the images of dendrites from channel one, channel two and their merging in CR. The synaptic boutons from the axons of piriform cortical neurons are found only in CR-formation mice.

**
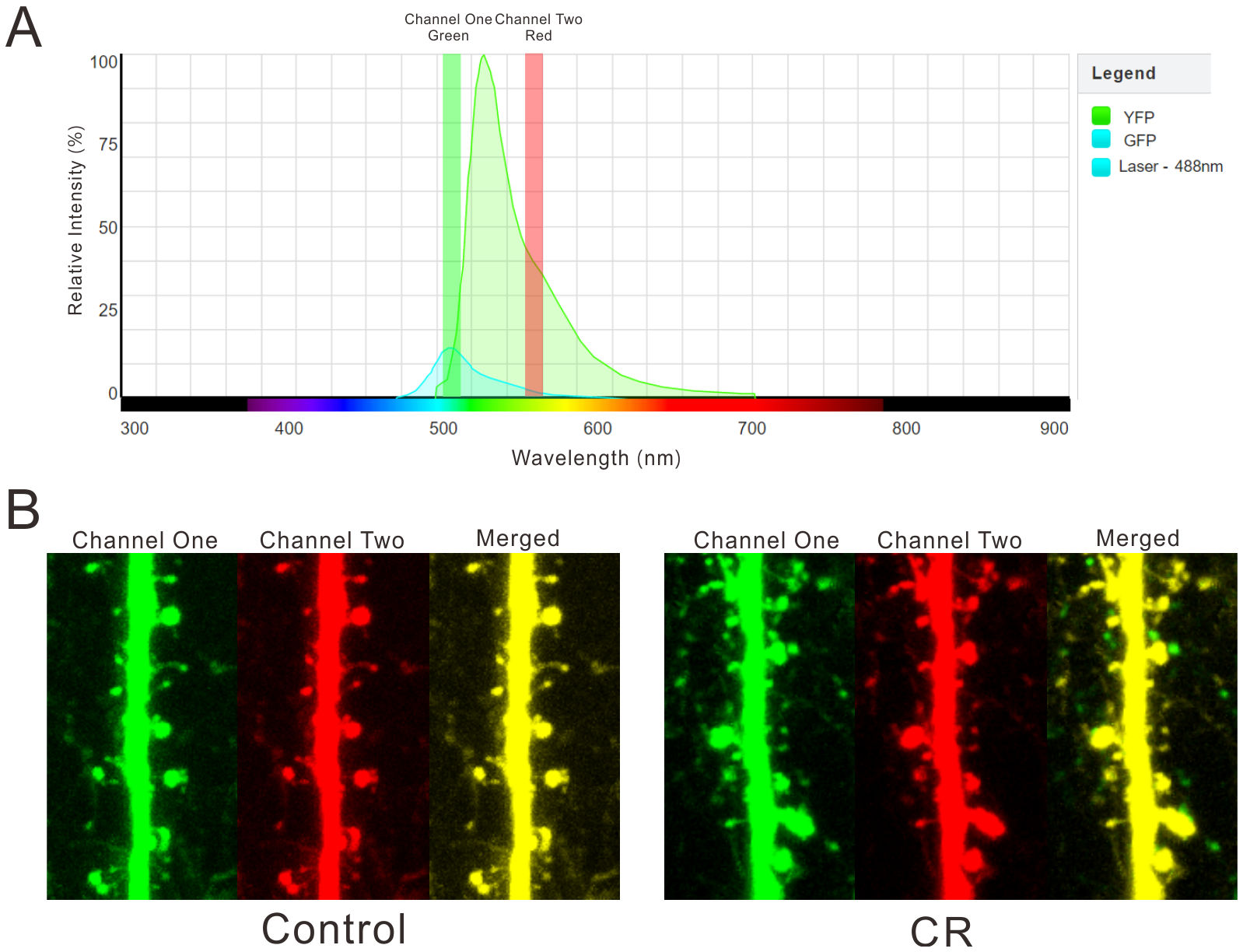
**
